# Association of cervical artery stenosis with common cerebral microvascular lesions and coronary artery calcifications

**DOI:** 10.3389/fnimg.2025.1559481

**Published:** 2025-06-27

**Authors:** Chiheb Louizi, Eya Khadhraoui, Joachim Lotz, Daniel Behme, Erelle Fuchs, Johannes T. Kowallick, Sebastian J. Müller

**Affiliations:** ^1^Institute of Diagnostic and Interventional Radiology, University Medical Center, Göttingen, Germany; ^2^Institute of Neuroradiology, University Medical Center, Göttingen, Germany; ^3^Clinic for Neuroradiology, University Hospital, Magdeburg, Germany; ^4^German Center for Cardiovascular Research DZHK, Göttingen, Germany; ^5^Goettingen Valley of Magnetic Resonance (GoeVMR), Göttingen, Germany

**Keywords:** microvascular lesion, Fazekas score, coronary artery calcification, cervical artery stenosis, atherosclerosis

## Abstract

**Background:**

A connection between cerebral white matter hyperintensities and coronary artery disease is widely discussed. Both conditions are more prevalent in the elderly. While white matter hyperintensities are often associated with small vessel disease, atherosclerosis is the primary cause of coronary artery disease.

**Methods:**

We evaluated staging CT scans of the body and staging brain MRIs from patients with newly diagnosed malignant melanoma (without metastasis) between 01/01/2015 and 06/30/2023. CT scans were assessed for coronary artery disease using a modified overall visual assessment. Fazekas scores were used to evaluate the MRI for white matter changes. Additional clinical data were obtained from digital patient records.

**Results:**

We analyzed data from 120 patients (57 females, mean age 68 years, standard deviation 14 years) and found a correlation between coronary artery disease and both age (*r* = 0.48, *α* = 0.04) and Fazekas score (periventricular r = 0.46, subcortical and deep white matter r = 0.55). A linear model including age, coronary artery disease, diabetes and arterial hypertension served as a predictor for white matter disease and showed significant correlations. Adding (1) atherosclerosis as well as (2) carotid stenosis to the model resulted in (1) a slight decrease in significance and (2) the unmasking of a potential spurious correlation with carotid stenosis.

**Conclusion:**

There is a significant correlation between white matter hyperintensities and both carotid stenoses and coronary artery disease. This finding is clinically relevant: in patients with white matter hyperintensities and coronary artery disease, carotid stenosis should be ruled out.

## Introduction

At first glance, cerebral microvascular lesions and coronary artery calcification (CAC) appear to have little in common. The etiology of microvascular lesions is heterogeneous, ranging from genetic to sporadic conditions, such as hypertension and chronic kidney disease, as well as the universal process of aging (Fang et al., 2023).

Coronary artery calcification, a marker of atherosclerotic cardiovascular disease is associated with several risk factors, including diabetes (Gadde et al., 2023), non-alcoholic fatty liver disease (Cucoranu et al., 2023), age, male sex, ethnicity (Lima Dos Santos et al., 2023), hypertension, sleep apnea (Castiglione et al., 2023), and cigarette smoking (Mallah et al., 2023).

Nevertheless, both frequently coexist. This raises the question of whether their co-occurrence is merely coincidental – such as in older patients - or whether cardiac conditions (e.g., untreated atrial fibrillation) my contribute to the development of brain lesions, or if a shared underlying vascular pathology exists. To explore this possibility, we conducted a statistical analysis to investigate potential associations. For this purpose, we selected patients with newly diagnosed malignant melanoma without distant metastases, who had no prior history of cardiac or cerebral disease and who had undergone brain MRI, chest and cardiac CT, as well as neck CT.

We chose this specific cohort because it provided simultaneous access to whole-body CT imaging—including coronary and cervical arteries—and high-resolution brain MRI, allowing for comprehensive vascular and neurological assessment.

**Figure 1:**
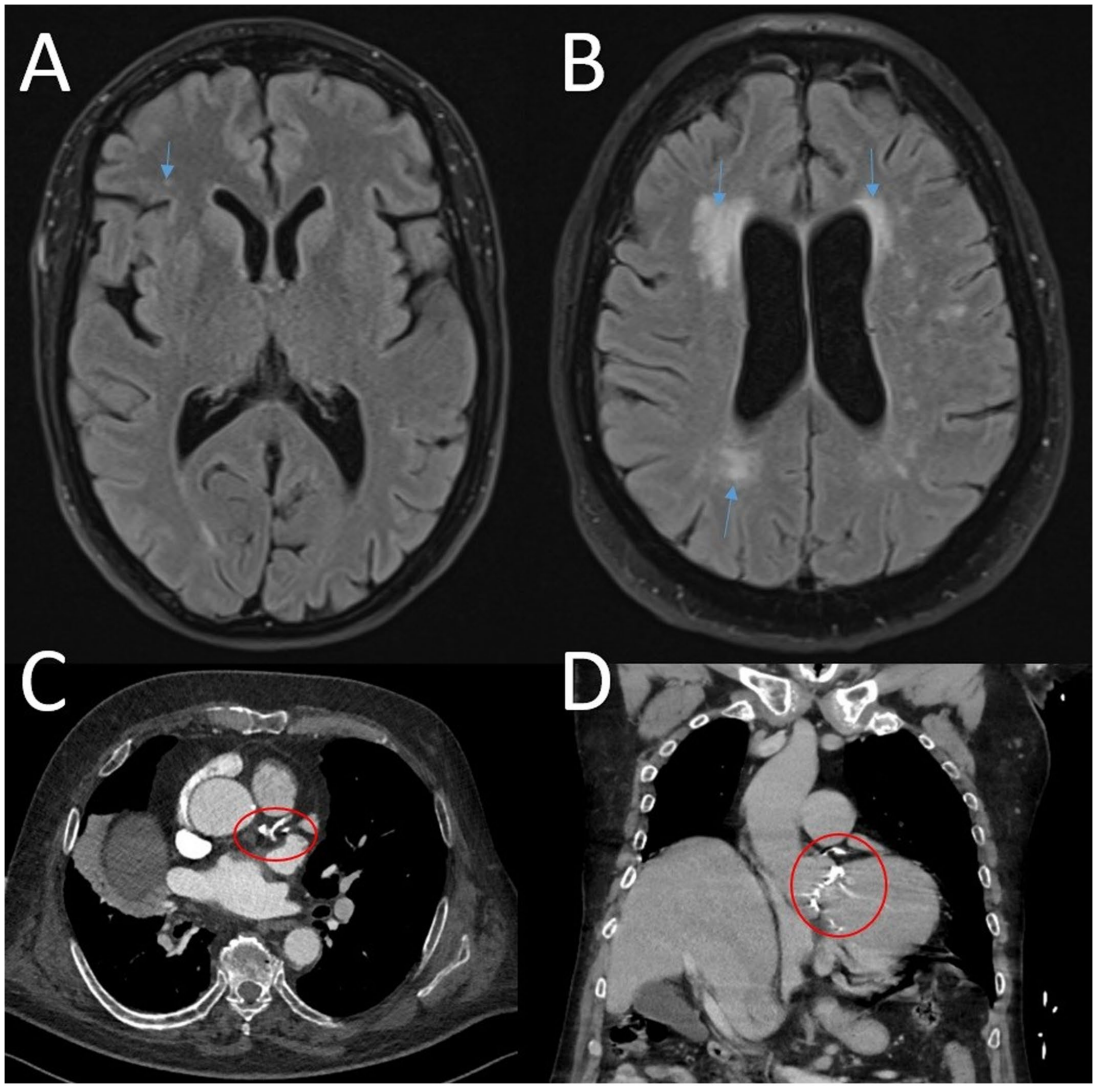
**(A,B)** MRI axial T2/FLAIR images of two patients. **(A)** Arrow points at single microvascular lesions (Fazekas score 1). **(B)** Arrows point at multiple confluent microvascular lesions (Fazekas score 2–3). **(C,D)** Contrast enhanced computed tomography scan showing severe coronary calcifications. **(C)** Axial. **(D)** Coronary.

Figure 1 provides representative examples and illustrates the main concepts of the Fazekas score and coronary artery calcifications.

## Methods

### Study design

A retrospective single-center observational study was conducted. Institutional review board approval from the Ethics Committee of University Medical Center Göttingen was obtained (Approval Number 27/8/23). Owing to the retrospective design of this study, the requirement for informed consent was waived by the ethics committee. All methods were carried out in accordance with relevant guidelines and regulations.

### Study population

We evaluated staging whole-body CT scans and brain MRIs from patients with newly diagnosed malign melanoma without metastasis.

To identify eligible cases, we searched our picture archiving and communication system (PACS) for patients with malignant melanoma who underwent brain MRI between 01/01/2015 and 06/30/2023.

Patients were excluded if they met any of the following criteria: absence of an in-house staging CT scan within 3 months of the MRI; presence of metastases at the time of imaging; age under 18 years; concurrent malignant or neurological disease—particularly multiple sclerosis; known heart disease other than coronary artery disease; history of radiotherapy; prior cardiac or brain surgery; or insufficient image quality.

### Image acquisition

MRI sequences from two different MR scanners were evaluated (1.5/3 Tesla, Siemens MAGNETOM Avanto and Prisma, Siemens AG, Werner-von-Siemens-Str. 1, D-80333 Munich, Germany). Transversal T2-/FLAIR sequences with a slice thickness of 4 mm and transversal T2 TSE sequences with a slice thickness of 2.5 mm were analyzed. Additional sequence parameters are provided in Table 1.

**Table 1:** MRI sequence details.

MRI	Sequence	Slice thickness	Resolution	TE	TR	TI
1.5 Tesla	T2w TSE tra	2.5 mm	0.9 mm × 0.9 mm	110 ms	4,700 ms	
	T2w-FLAIR tra	4 mm	0.5 mm × 0.5 mm	117 ms	10,000 ms	2,600 ms
3 Tesla	T2w TSE tra	2.5 mm	0.5 mm × 0.5 mm	108 ms	3,000 ms	
	T2w-FLAIR tra	4 mm	0.5 mm × 0.5 mm	96 ms	9,000 ms	2,500 ms

TSE, turbo spin echo; FLAIR, fluid inversion recovery; TE, echo time; TR, repetition time; TI, inversion time.

All subjects also underwent contrast-enhanced CT imaging (SOMATOM AS+, FLASH and FORCE, Siemens Healthineers, Erlangen, Germany). The CT images had a slice thickness of 0.625–1.5 mm. All CT examinations included the portal venous phase, using 100 mL of iodine-based contrast agent (minimum 350 mg iodine/ml) with a 60-s delay.

### Imaging analysis

The imaging analysis included (Fang et al., 2023) an evaluation of the T2-weighted/FLAIR—sequence of the brain, and (Gadde et al., 2023) an assessment of the staging CT of the neck, chest, abdomen, pelvis.

According to the Fazekas score (Fazekas et al., 1987), lesions in the deep white matter (DWM) and periventricular white matter (PVWM) were visually assessed in the T2-weighted/FLAIR – sequence.

**Figure 2:**
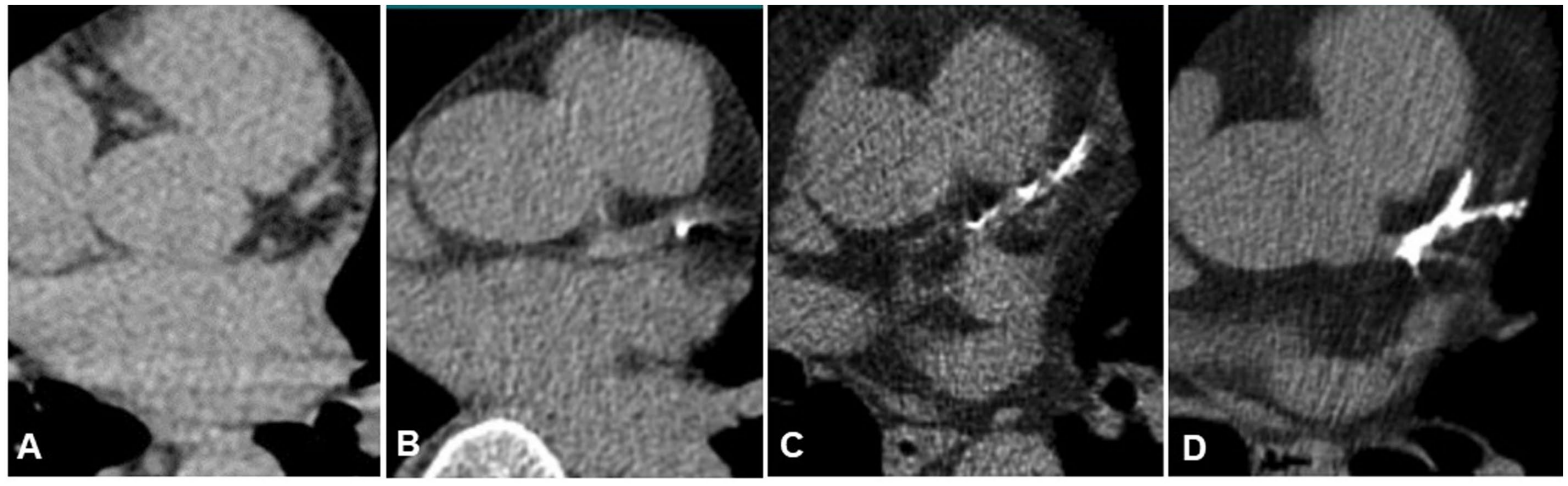
**(A)** Example of none CAC (score of 0). **(B)** Example of mild CAC (score of 1). **(C)** Example of moderate CAC (score of 2). **(D)** Example of severe CAC (score of 3).

For CAC in the CT scan, an overall visual assessment (Chiles et al., 2015) scoring system was used. Figure 2 illustrates examples of none, mild, moderate, or severe coronary artery calcifications (0–3): no-CAC = 0, mild-CAC = 1, moderate-CAC = 2, severe-CAC = 3. The right coronary artery (RCA), left main and left anterior descending artery (LCA-LAD) and left circumflex artery branch (RCX) were evaluated. The four CAC scores from these territories were summed to create a modified overall visual assessment score (MOVAS), ranging from 0 to 12. Patients with coronary stents or coronary artery bypass grafts were excluded. Due to the retrospective nature of the study, quantitative Agatston scoring was not feasible, as ECG synchronization was unavailable.

Additionally, we assessed the presence of cervical calcifications of the right and left carotid bifurcation (0–1; 0—no calcifications; 1—calcifications) and stenosis of the inner carotid artery (qualitative assessment; 0–3; 0—no stenosis; 1—mild stenosis; 2—moderate stenosis; 3—severe stenosis).

All assessments were performed independently by two radiologists (CL and EK), who evaluated the T2-/FLAIR-weighted brain images and staging CT scans (neck, chest, abdomen, pelvis) using the GE Centricity^™^ Universal Viewer (GE Healthcare, 500 W Monroe St, Chicago, IL 60661, United States).

The measured values were used to calculate intra-class correlation coefficients (ICCs). Any discrepant cases were reviewed jointly, and a consensus value was agreed upon in each instance.

### Statistical analysis

Statistical analyses were conducted using Statistica, version 13 (TIBCO Software Inc., Palo Alto, CA, United States) with a significance level set at *p* < 0.05.

Basic statistics, including standard deviation and Pearson’s correlation coefficient, were used to summarize the data. We evaluated odds ratios (Szumilas, 2010) of Fazekas Score/coronary calcifications and cervical artery stenosis.

Multilevel linear models were performed using the “lm” function in R Version 4.2.2.^1^ Tukey’s test (Tukey, 1949) was used to compare the means of Fazekas subgroups with respect to coronary artery calcifications.

The term spurious correlations refers to statistical associations between variables that arise not due to a direct causal relationship, but rather due to the influence of one or more confounding variables. In the context of our analysis, we observed such potentially misleading correlations—particularly between cerebral microvascular lesions, coronary artery calcifications, and cervical stenosis. To address this, we tested for multicollinearity using the Variance Inflation Factor (VIF) (Kim, 2019), which allowed us to assess the degree of redundancy among predictors in our linear models. We calculated the VIF using the “regclass” library of R. The VIF(*j*) for a given predictor is defined as: 1/(1 − *R*^2^(*j*)), where *R*^2^ is the coefficient of determination obtained from a regression of that predictor against all other independent variables in the model. *R*^2^ with confidence intervals was calculated with the “lm” function and the “mbess” library of R. A high VIF indicates that the variable is highly collinear with other predictors, which can inflate the variance of coefficient estimates and reduce model reliability. Common thresholds suggest that a VIF greater than 5 may indicate problematic multicollinearity. Additionally, we assessed inter-rater reliability using the intraclass correlation coefficient (ICC) in R, utilizing the irr, readxl, lpSolve, and psych packages. The ICC was calculated based on a mean-rating, absolute-agreement, two-way random-effects model. Interpretation of ICC values followed the guidelines proposed by Koo and Li (2016).

## Results

### Study population

A cohort of 120 patients (57 females, 63 males) with a mean (± standard deviation) age of 68 ± 14 years at the time of cranial MRI was identified. Baseline characteristics were distributed as expected and are summarized in Table 2.

**Table 2:** Base parameters of the patient cohort.

Parameter	All	Female	Male
Count (*n*=)	120	57	63
Mean age (± standard deviation)	68 ± 14	68 ± 15	68 ± 13
Mean size (± standard deviation)	171 ± 9 cm	165 ± 6 cm	176 ± 7 cm
Mean weight (± standard deviation)	79 ± 17	72 ± 15	86 ± 17
Mean BMI (± standard deviation)	27 ± 5	27 ± 5	28 ± 4
Diabetes (*n*=)	13	5	8
Arterial hypertension (*n*=)	67	27	39

BMI, body mass index.

Nicotine abuses could not be reliably assessed in all cases due to imcomplete documentation in the records and was therefore excluded from statistical analysis.

### White matter hyperintensities

**Figure 3:**
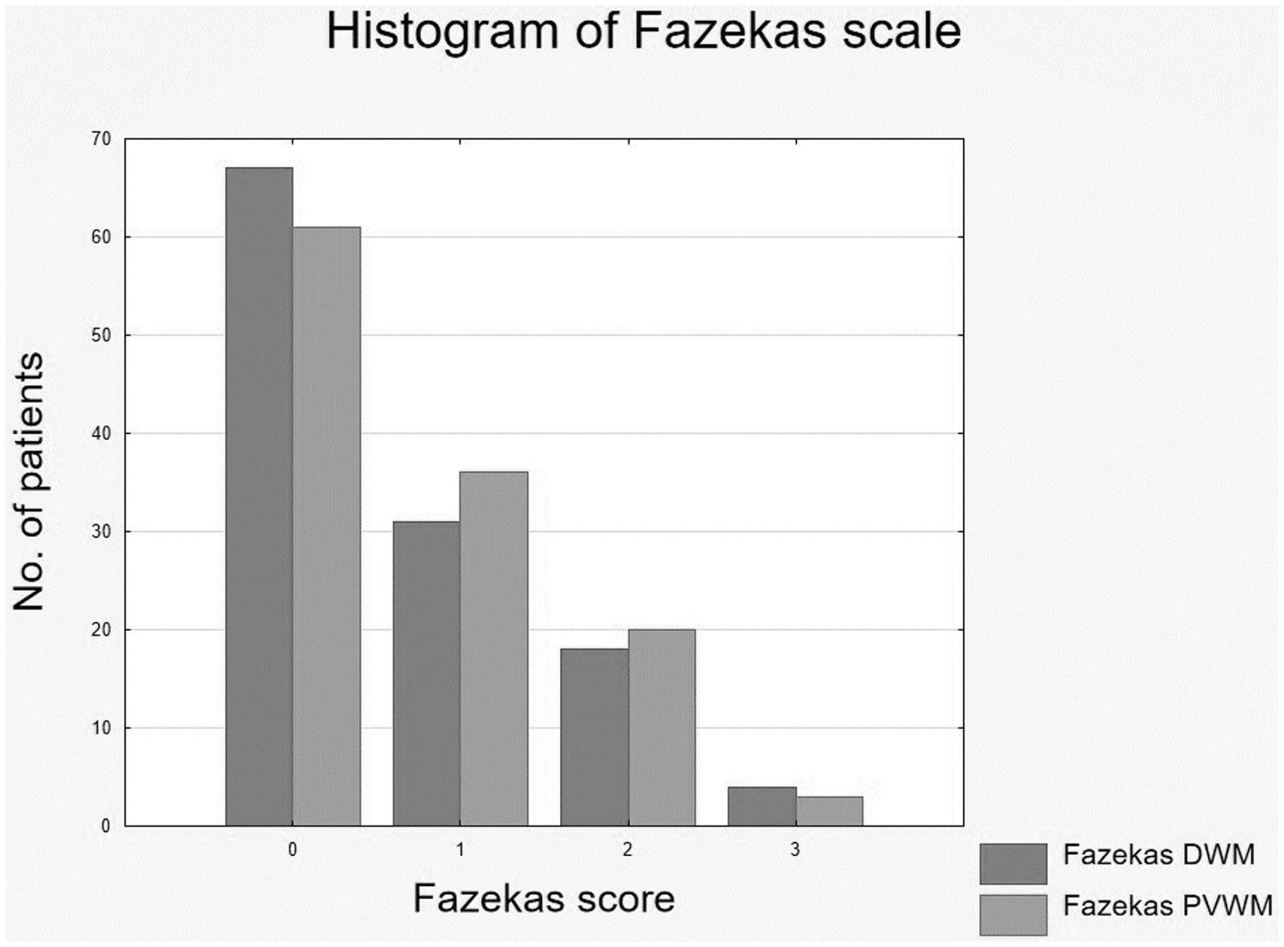
Distribution of measured white matter hyperintensities following Fazekas. DWM, deep white matter; PVWM, periventricular white matter.

White matter hyperintensities were identified in 64 of the 120 patients. DWM lesions were present in 53 patients, and PVWM lesions were found in 59 patients. In most cases—31 for DWM and 36 for PVWM—only mild changes (Fazekas 1) were observed. A frequency distribution is presented in Figure 3.

### Coronary artery calcifications

**Figure 4:**
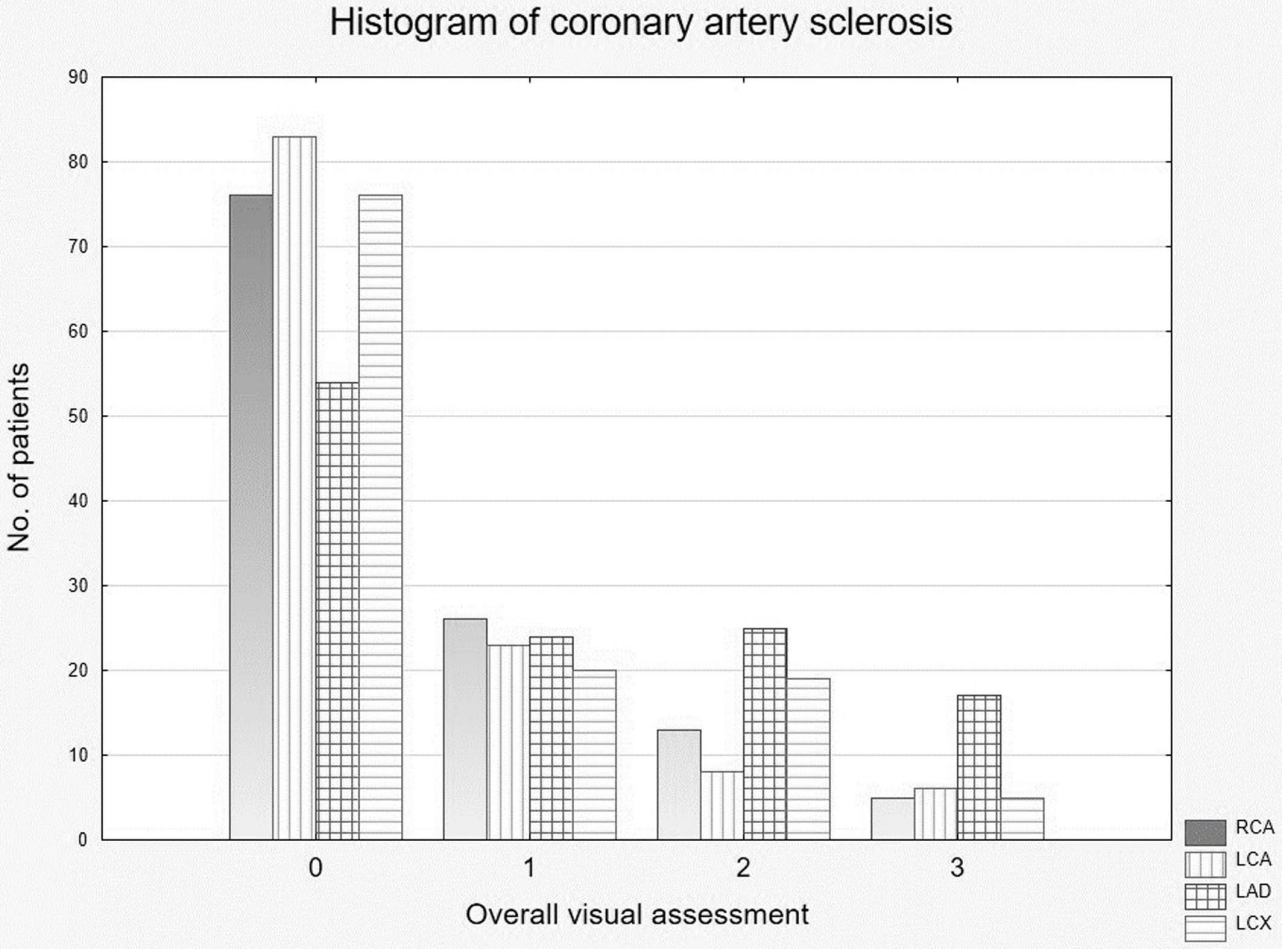
Distribution of measured (single vessel) coronary artery calcifications. RCA, right coronary artery; LCA, left coronary artery; LAD, left anterior descending artery; RCX, ramus circumflex artery.

CAC were detected in 67 of the 120 patients. Table 3 presents the results of the MOVAS (0–12) as a sum of the single scores of the four main vessels (RCA, LAD, LCA, RCX). A detailed frequency chart for each vessel is shown in Figure 4.

**Table 3:** Distribution of the modified overall visual assessment score (0–12) of the coronary arteries.

MOVAS	0	1	2	3	4	5	6	7	8	9	10	11	12
Patients (*n*=)	67	9	11	11	5	3	10	3	4	4	4	2	1

MOVAS, modified overall visual assessment score, sum of the single scores of the three vessels (RCA, LAD, LCA, RCX).

### Cervical stenosis

Neck CT scan were available for all 120 patients. A correlation was observed between cerebral white matter lesions and the presence of arteriosclerotic plaques.

We detected carotid stenoses in 21 patients, mild stenoses in seven patients, and moderate stenoses in 13 patients. Only in one patient was a severe stenosis detected. In 13 patients (five only left-sided, three only right-sided) a stenosis was found on both sides.

The odds ratio (OR) for having a cervical stenosis in patients with a Fazekas score (sum) > 0 (*n* = 64) was 6.5. For patients with a MOVAS > 0 (*n* = 67), the OR was 29.0. Patients with both a Fazekas score > 0 and MOVAS > 0 (*n* = 54) had an OR of 9.9 for cervical stenosis.

### ANOVA analysis

Tukey’ test, as shown in Table 4, revealed significant differences in coronary artery calcifications across the cerebral microvascular lesion subgroups (Fazekas scores).

**Table 4:** Tukey’s test (Tukey multiple comparisons of means 95% family-wise confidence level) of modified overall visual assessment score of the coronary arteries for the Fazekas subgroups.

Compare (MOVAS)	Diff	Lower	Upper	*p*
Fazekas 0 vs. Fazekas 1	1.61	0.33	2.89	**0.007**
Fazekas 0 vs. Fazekas 2	2.97	1.48	4.47	**0.00001**
Fazekas 0 vs. Fazekas 3	4.76	1.63	7.90	**0.0007**
Fazekas 1 vs. Fazekas 2	1.36	−0.25	2.96	0.12
Fazekas 1 vs. Fazekas 3	3.16	−0.03	6.34	**0.05**
Fazekas 2 vs. Fazekas 3	1.79	−1.49	5.07	0.48

Bold—significant with *p* < 0.05; MOVAS, modified overall visual assessment score.

### Interrater agreement

The interrater reliability for the assessment of microvascular lesions (Fazekas score) was good to excellent with an ICC estimate of 0.90 and a 95% confidence interval of 0.80 to 0.95.

The rating of the coronary artery calcifications (modified overall visual assessment) demonstrated an excellent ICC of 0.96 with a 95% confidence interval of 0.91 to 0.98.

The assessment of the cervical arterial calcifications and stenoses demonstrated an excellent ICC of 0.93 with a 95% confidence interval of 0.86 to 0.96.

### Simple correlations

Pearson’s correlation matrices revealed significant associations between the Fazekas scale with age, body height (negative correlation), arterial hypertension, diabetes, coronary artery calcifications and cervical artery calcifications. Coronary artery calcifications further correlated with age, sex, arterial hypertension, diabetes and cervical artery calcifications. The matrices are shown in Tables 5, 6.

**Table 5:** Correlation matrix between Fazekas scales and other parameters revealed several significant correlations (*n* = 120; bold correlation coefficient *r*; *p* < 0.05).

Correlation coefficient r	Age	Sex	Height	Weight	BMI	Art. hypertension	Diabetes	MOVAS	RCA	LAD	LCA	RCX	CAP	CS
Fazekas sum	**0.56**	0.03	**−0.21**	−0.10	0.02	**0.24**	**0.29**	**0.44**	**0.41**	**0.44**	**0.32**	**0.37**	**0.40**	**0.37**
Fazekas DWM	**0.50**	−0.01	**−0.20**	−0.07	0.05	**0.21**	**0.27**	**0.41**	**0.40**	**0.40**	**0.33**	**0.34**	**0.36**	**0.38**
Fazekas PVWM	**0.58**	0.07	**−0.20**	−0.13	−0.02	**0.25**	**0.28**	**0.43**	**0.39**	**0.47**	**0.29**	**0.37**	**0.41**	**0.32**

Bold—significant with *p* < 0.05; MOVAS, modified overall visual assessment score; RCA, right coronary artery; LAD, left anterior descending artery; LCA, left coronary artery; RCX, ramus circumflex artery; CAP, cervical arteriosclerotic plaques; CS, cervical arterial stenosis; DWM, deep white matter; PVWM, periventricular white matter.

**Table 6:** Correlation matrix between coronary artery calcifications and other parameters revealed several significant correlations (n = 120; bold correlation coefficient r; p < 0.05).

Correlation coefficient r	Age	Sex	Height	Weight	BMI	Art. hypertension	Diabetes	CAP right	CAP left	CS right	CS left
MOVAS	**0.47**	**0.25**	−0.06	0.02	0.07	**0.38**	**0.35**	**0.47**	**0.50**	**0.59**	**0.58**
RCA	**0.44**	**0.15**	0.05	0.09	0.14	**0.42**	**0.31**	**0.42**	**0.43**	**0.56**	**0.54**
LAD	**0.48**	**0.27**	−0.05	−0.02	0.05	**0.36**	**0.34**	**0.47**	**0.48**	**0.55**	**0.56**
LCA	**0.35**	**0.24**	−0.06	0.02	0.07	**0.20**	**0.24**	**0.39**	**0.42**	**0.46**	**0.41**
RCX	**0.41**	**0.27**	−0.06	−0.04	−0.04	**0.27**	**0.35**	**0.39**	**0.45**	**0.55**	**0.54**

Bold—significant with *p* < 0.05; MOVAS, modified overall visual assessment score; RCA, right coronary artery; LAD, left anterior descending artery; LCA, left coronary artery; RCX, ramus circumflex artery; CAP, cervical arteriosclerotic plaques; CS, cervical arterial stenosis.

The truth of these correlations has not proven as it could be spurious correlations.

### Linear models without cervical atherosclerosis and carotid stenosis

To account for potential spurious correlations, we constructed multiple linear models using the “lm”-function in R. We first tested a basic model to assess whether age confounds the relationship between the sum of the Fazekas scores (FAZEKAS_sum) and coronary artery calcifications (MOVAS). The model used was “lm (FAZEKAS_sum ~ AGE + MOVAS, data = data).” Results are presented in Table 7, showing that the significant association between FAZEKAS_sum and MOVAS remained after adjusting for age.

**Table 7:** Results of the call “lm (FAZEKAS_sum ~ AGE + MOVAS, data = data)” in R (*n* = 120, adjusted *R*^2^ = 0.33, 95%-confidence-internal 0.19–0.47, residuals median −0.23; 1Q −0.85; 3Q 0.58).

R output	Estimate	Standard error	*t* value	Pr (>|t|)	VIF
(Intercept)	−0.881	0.314	−2.805	**0.006**	
AGE	0.020	0.005	4.165	**0.000006**	1.28
MOVAS	0.060	0.023	2.627	**0.010**	1.28

Bold—significant with *p* < 0.05; MOVAS, modified overall visual assessment score; |*t* value| >2 and Pr (>|*t*|) < 0.05—significant. *R*^2^, coefficient of determination; VIF, variance inflation factor; 1Q, first quartile; 3Q, third quartile.

Next, we applied a more comprehensive model to control for additional clinical variables, particularly arterial hypertension (AHP) and diabetes (DIAB): “lm (formula = FAKEKAS_sum ~ AGE + MOVAS + BMI + AHP + DIAB, data = data).” As shown in Table 8, including or excluding body mass index (BMI), height, or weight did not significantly alter the results. These findings suggest that arterial hypertension and diabetes are not independently associated with FAZEKAS_sum but likely act as confounding variables through their association with coronary artery disease and/or systemic atherosclerosis.

**Table 8:** Results of the call “lm (formula = FAZEKAS_sum ~ AGE + MOVAS + BMI + AHP + DIAB, data = data)” in R (*n* = 120, adjusted *R*^2^ = 0.33, 95%-confidence-internal 0.20–0.47, residuals median −0.25; 1Q −0.87; 3Q 0.73).

R output	Estimate	Std. error	*t* value	Pr (>|*t*|)	VIF
(Intercept)	−1.900	0.953	−1.994	**0.049**	
AGE	0.046	0.009	4.963	**0.000002**	1.38
MOVAS	0.096	0.044	2.176	**0.032**	1.45
BMI	−0.002	0.027	−0.091	0.930	1.09
AHP	−0.124	0.285	−0.441	0.660	1.36
DIAB	0.715	0.428	1.671	0.097	1.22

Bold—significant with *p* < 0.05; MOVAS, modified overall visual assessment score; BMI, body mass index; AHP, arterial hypertension; DIAB, diabetes; |*t* value| > 2 and Pr (>|*t*|) < 0.05—significant. *R*^2^, coefficient of determination; VIF, variance inflation factor; 1Q, first quartile; 3Q, third quartile.

### Linear models including cervical artery calcifications and stenosis

Because coronary artery calcifications (MOVAS), cervical atherosclerotic plaques (CAP), and carotid stenosis (CS) are interrelated, using them as independent variables within the same linear model is statistically limited. Nevertheless, we explored this interaction with the following model: “lm (formula = FAZEKAS_sum ~ AGE + MOVAS + BMI + AHP + DIAB + CAP + CS, data = data).”

As expected, the results (shown in Table 9) were inconsistent and produced paradoxical outcomes. In contrast, a simplified model: “lm (formula = FAZEKAS_sum ~ MOVAS + CAP, data = data)” revealed significant correlations for both MOVAS and CAP. Notably, adding CAP to more complex models did not eliminate the significance of MOVAS.

**Table 9:** Results of the call “lm (formula = Fazekas_Sum ~ AGE + MOVAS + AHP + DIAB + CAP left + CAP right + CS left + CS right, data = data)” in R (*n* = 80, adjusted *R*^2^ = 0.35, 95%-confidence-internal 0.19–0.50, residuals median −0.20; 1Q −0.85; 3Q 0.83).

R output	Estimate	Std. error	*t* value	Pr (>|*t*|)	VIF
(Intercept)	−1.899	0.569	−3.335	**0.001**	
AGE	0.042	0.010	4.269	**0.0004**	1.66
MOVAS	0.042	0.054	0.776	0.440	2.24
AHP	−0.158	0.274	−0.577	0.565	1.31
DIAB	0.610	0.428	1.428	0.156	1.25
CAP left	−0.182	0.353	−0.617	0.606	2.20
CAP right	0.554	0.363	1.526	0.130	2.26
CS left	0.578	0.316	1.825	0.071	2.86
CS right	−0.239	0.358	−0.669	0.505	2.85

Bold—significant with *p* < 0.05; MOVAS, modified overall visual assessment score; AHP, arterial hypertension; DIAB, diabetes; CAP, cervical arteriosclerotic plaque (left + right); CS, cervical stenosis (left + right); |*t* value| > 2 and Pr (>|*t*|) < 0.05—significant. *R*^2^, coefficient of determination; VIF, variance inflation factor; 1Q, first quartile; 3Q, third quartile.

However, when CS was added to models containing both FAZEKAS_sum and MOVAS, the previously significant correlation between MOVAS and FAZEKAS_sum disappeared. This suggests that carotid stenosis may mediate the observed association between coronary calcifications and white matter lesions.

Further model testing showed that removing MOVAS or right-sided carotid stenosis from the model restored a significant association between FAZEKAS_sum and left-sided stenosis (*p* = 0.048 for both models). Conversely, removing left-sided stenosis and retaining right-sided stenosis resulted in a non-significant association (*p* = 0.067).

The analysis suggested moderate multicollinearity, with VIF values ranging from 1.1 to 2.9.

A summary of the key findings is listed in Table 10.

**Table 10:** Summary of key findings of the multivariate models.

Parameter	Coronary artery calcifications	Cervical stenosis	White matter hyperintensities
Coronary artery calcifications	X	X	(X)
Cervical stenosis	X	X	(X)
White matter hyperintensities	(X)	(X)	X
Age	X	X	X
Sex	X		
BMI			
Height			(−) X
Weight			
Arterial hypertension	X	X	(X)
Diabetes	X	X	(X)
Cervical plaques	X	X	(X)

X—significant positive correlation at a significance level of 0.05; (−) X—significant negative correlation; (X)—significant correlation dependent on linear model.

## Discussion

In this study, we confirmed a close relationship between cerebral microvascular lesions, a left-sided inner carotid artery stenosis and coronary artery calcifications. In contrast, the mere presence of cervical arteriosclerotic plaques alone did not appear to have a significant impact. These findings suggest that additional, yet unidentified, factors may contribute to a shared predisposition toward vascular stenosis across large, medium, and small vessels.

Cerebrovascular brain lesions are one of the most common (“incidental”) findings in cranial MR imaging. Fazekas introduced a scoring of “white matter hyperintensities” (WMH) and periventricular medullary lesions (PVH) (Fazekas et al., 1987). Today, such lesions should not be viewed as normal aging, but as symptoms of another underlying disease (Statsenko et al., 2022). Arterial hypertension, stroke (Sperber et al., 2024) and atherosclerosis are mainly suspected (Jellinger, 2002), but other reasons such as genetic (Yektay Farahmand et al., 2023), constitutional (Choi et al., 2023), nutritional (Zhang et al., 2021) or environmental cause (Uretsky et al., 2022), are also discussed.

It is clear that the lesion burden increases with age and is significantly increased in cerebrovascular disease (CVD) (Awad et al., 1987). Arterial hypertension (van Dijk et al., 2004) also seems to play a significant role, while other vascular risk factors such as diabetes, gender and coronary artery disease were not considered important in previous studies (Awad et al., 1986). Iidaka (1993) found no significant connection between ischemic heart disease and cerebral lesion burden in a Japanese study. In contrast, Choi et al. (2022) found an association between cerebral white matter hyperintensities with coronary artery calcifications in 1,337 healthy individuals.

A significant association was also detected between arteriosclerotic changes of the neck vessels and cerebral lesions (Kim et al., 2014); in this study only patients without stenosis were included. Ironically, as part of a multifactorial analysis of this study, arterial hypertension correlated with the presence of arteriosclerosis, but not with cerebral microvascular lesions (“apparent correlation”).

In another study, a strong association was found between intracranial atherosclerotic stenosis and hypointense white matter lesions (Park et al., 2015). A comparative study found that there was a significant increase in lesion burden with intracranial as compared to extracranial atherosclerotic plaque burden (Lee et al., 2011).

There are also strong differences between untreated and treated arterial hypertension (Fukuda and Kitani, 1995), demonstrating the preventive nature of antihypertensive medication for cerebral lesions. Nicotine abuse also seems to play an important role (Fukuda and Kitani, 1996).

While smoking is a well-established risk factor for atherosclerotic changes across various vascular territories, including coronary (Elo-Eghosa et al., 2025), cerebral (Ingall, 1991), and extracranial carotid arteries (Babiker, 2016), the differential impact of smoking on the interrelationships among CAC, WMH, and cervical stenosis remains underexplored. Some studies suggest that smoking may amplify the severity of these vascular changes, potentially leading to stronger associations among these markers in smokers compared to non-smokers (Hou et al., 2019). However, the existing literature lacks definitive evidence to confirm whether the correlations between CAC, WMH, and cervical stenosis differ significantly between smokers and non-smokers. Further research, particularly prospective studies with stratified analyses by smoking status, is necessary to elucidate these potential differences.

An examination of the distribution of lesions according to age and disease showed that in patients with arterial hypertension, the subcortical and deep lesions are more frontal and appear in the fifth decade of life, while cognitive decline occurs in later years together with parieto-occipital periventricular lesions (Habes et al., 2018). To our knowledge, a precise correlation between atherosclerotic changes of the coronary arteries and these lesions has not yet been established. There are multiple methods for evaluating coronary artery disease [overall visual assessment, segmented vessel-specific scoring (Chiles et al., 2015), Agatston scoring (Agatston et al., 1990; Janowitz et al., 1991; Arad et al., 2000)], of which we used a modified overall visual assessment (Chiles et al., 2015). We chose this score due to the retrospective nature of the study with missing ECG triggers and contrast enhanced CT scans.

A strong correlation between vessel disease of heart and brain has been described in several genetic diseases, such as cerebral autosomal dominant and recessive arteriopathy with subcortical infarcts and leukoencephalopathy; CADASIL (Argirò et al., 2021) and CARASIL (Müller et al., 2020), respectively—as well as moyamoya-like cerebrovascular diseases (Tokunaga et al., 1996). The concept of a link between cardiovascular pathology and cerebral small vessel disease is not new (Andin et al., 2005). However, growing evidence supports the hypothesis that small vessel disease represents a systemic vascular disorder (Feuer et al., 2022; Mazini et al., 2023). While the association between coronary microvascular dysfunction and renal failure is well-established (Nelson et al., 2019), the relationship between chronic kidney disease and cerebrovascular pathology has only recently gained attention (Marini et al., 2021).

### Limitations of the study

This study has several limitations, primarily due to its retrospective, single-center design. Notably, reliable data on cigarette smoking status were unavailable for all patients, limiting our ability to include this important cardiovascular risk factor in the analysis.

The sample size was relatively small, which may affect the generalizability of the findings. Additionally, the use of contrast-enhanced venous-phase CT scans poses limitations for accurately grading both carotid stenoses and coronary artery calcifications.

Another limitation is that both coronary calcifications and carotid stenosis were assessed qualitatively rather than quantitatively. However, this approach reflects routine clinical practice and was applied consistently across all subjects. Incorporating quantitative measurement methods in future studies may enhance the precision and robustness of statistical analyses.

Furthermore, we must question whether it is enough to look for calcium, or does one have to look for the precursor—the atheromatous plaque, since calcification is the healed form of the plaque and is therefore more likely to be seen as a long-term consequence of the underlying disease (Fleg et al., 2012).

Lastly, the observed association between left-sided cervical stenosis and cerebral microvascular lesions may be incidental, reflecting the random distribution of stenoses in our cohort. Previous studies have not reported a consistent left-sided predominance in this context (Yasin and Tasdemir, 2023), suggesting that this finding should be interpreted with caution.

### Outlook

Emerging MRI techniques, such as quantitative mappings (Wang et al., 2018; Müller et al., 2022), may enhance the differentiation of cerebral lesions und assist in identifying their underlying causes, which range from genetic to sporadic (e.g., hypertension, chronic kidney disease), as well as age-related changes.

Quantitative vessel and stenosis measurements using cardiac computed tomography and state of the art cervical angiography would also be necessary for further prospective studies to untangle the network of countless co-factors. The potential interdependence of variables such as diabetes, hypertension, and atherosclerotic plaque burden remains an area of active debate.

Based on our observations in this small cohort of melanoma patients, the co-occurrence of cerebral microvascular lesions and coronary artery calcifications should prompt clinicians to consider the possibility of additional artery stenoses. These may include cervical artery stenosis (Fanning et al., 2006), as demonstrated in our study, as well as stenoses in other vascular territories reported in the literature—such as the celiac trunk, superior mesenteric artery (Krishnamurthy et al., 2019), renal arteries (Joosen et al., 2012; Kataoka et al., 2009), and in the context of peripheral artery disease (Meer et al., 2024).

## Conclusion

Our study provides initial evidence for a correlation between cerebral microvascular lesions and coronary artery calcifications. While the presence of cervical atherosclerotic plaques did not significantly affect the results of our linear models, the presence of cervical artery stenosis had a marked influence.

This finding may reflect an increased risk of recurrent cerebral micro-emboli associated with stenosis. Alternatively, it may indicate shared underlying etiologies—such as genetic predisposition, cigarette smoking, or environmental factors—that contribute to the development of cerebral microvascular lesions, coronary artery calcifications, and cervical stenosis. These possibilities echo the hypothesis proposed by Fazekas et al. (1988) over three decades ago, suggesting that vascular changes across different organ systems may reflect a common systemic process.

## Data Availability

The original contributions presented in the study are included in the article/supplementary material, further inquiries can be directed to the corresponding author.

## Footnotes

^1^ https://r-project.org/
